# Supplementary material for: Chromothripsis during telomere crisis is independent of NHEJ, and consistent with a replicative origin
Source: Genome Res. 2019 May;29(5):737–49. doi: 10.1101/gr.240705.118 (PMC6499312; doi:10.1101/gr.240705.118)
Supplement: Supplemental Material [file supp_gr.240705.118_Supplemental_file_1.zip › contigs/annotated_contigs/DB57/contig.5.DB57_length_1559_mean_cov_19.9146889031.docx]

**DB57_length_1559_mean_cov_19.9146889031**

```
AAACCAGAAACCAAGAAAGAAACAAACAAAAGTAAAAAA|AAAAAAGTGTAAATATCCATAGAGACAGCAATCAGTAAATACGTGAAGT
                                        >chr7:141699954-141700481 - E=1e-304
AAAATATAATTCAATGTTATTTAAAATCATCACACGGCAGGTGTTTTTAATTTAACAAATTATTTGATACTCCTCTCTTCAAGAGGTAG

GGCGAGTCCCCACCACCTCACCCTCTGAGGATGAGCTGGACTTAGTGACTCACTTTCAACAGATAGGATGTGGAGAAGGGATGGCATCT

AACTTCCAAGACTAGGCATTACAGCTTCCTCCTTGCTCTTTCTCTTGGATCATTTCCTGAAGAAACCAGTTACCATGCCATCATAACAT

TCAAGCAGGACTATGGAAATTTCTAGCAAAGAACTGAGGTCTTCTGCTAAGAGCCATCTGAGTGAGGTCCCTTGAAAGCAAGCCTCTAC

CCCTATTCAAGCCTTCACATGACTACAGTCCTGGCTGACATATTGGCTGGATTCATGAGAGACCCTAAATCAGCACCACTCGGCTAACT

CACCCCCATATTTCTGACCTACATAAATTGGGA|CTTGTAAATTTACAAG|TATAAAGGAAAATTCAGTGACTTGGAGATTTGAGGTTT
                                                  >chr7:141740897-141741034 - E=3e-70
GCATAAGGTAATTTTTGAATCACAAGAAAGAAATCTTAGTGGGAGAAAAGAAATAGGAAGTTGAAATTCTAGGAGGGAGGAGAGTCAAG

GTTCTCTAGA|CTTCCTTTATACA|GGCTCATGCCTGTAATCCCAGCACTTTGGGAGGCTGAGGCATGTGGATCACGAGGTCAGGACCT
                        >chr19:40463158-40463378 + E=4e-120
CAAGACCAGTCTGGCCAACATGGTGAAACCCCGTGTCTACTAAAAATACAAAAATTAGCTGGGCATGGTGGCACGCATCTGTAATCCCA

GCTGCTCGGGATTACAAAAAACAAGAACAGCTAAGAAAGTTCTGAAAAACTCAGGAGGCTGAGGCAA|TCACATCCCTTTTGTCCAATC
                                                                     >chr10:931213-93132
ATGCTTCTCCACAACTGTCAACATCTGCATTAGACTTCGCAAAAAAAAAATACAGTTTTCCCTGTTTCTTTGGTTCTTCCTTTGTGG|A
3 + E=5e-54
G|TGACTAGATCCTTCTTATAAATGCTCTTCAGGTAGCCCAAAGTTCTCCTTTGTATAACCCATTATAATTAGATTATACATATAATTG
>chr7:137422702-137423172 - E=2e-264
TGTAGAGGTATGTATTTAATATGTGTCTCTCCTAGTAGCTTGGAGTCAGTATAGCACAGCAGCTAAGGGTCCAGCCCTCGAGATCTGCT

TTCAAATTCTACTTTTGTCACTTAGTATCTACATTACACCGATTAACTTAATCAGTGTCATGTGGTCCTGCTAACTGCATTAATTTGTC

TGTATTTCATTCCGCATCTACAAAATGGTAATAAACATAGGACATATTTTATAGGGCTATATATTGAATGTATTAAATAAGAAAATCCT

TGAGTATTGCTTAGCACTGTGCCTAGAACATGAAAACTCTTAATAAAGCTTGGCTATTAGTGTTGCTAAACGGTAAAGACTGTTTCTGT

CTTATCCCTTGTGTTATCCCCAGTT|GTTTGGAGAGGACGTGTTGCACGCAGATG
```
